# Spatiotemporal patterns of prevalence and mortality from respiratory infections and tuberculosis across Japan and its prefectures

**DOI:** 10.1371/journal.pone.0351936

**Published:** 2026-06-17

**Authors:** Xiaohui Li, Gang Wu, Zhimin Ding

**Affiliations:** 1 Department of Infectious Diseases, Dazhou Central Hospital, Dazhou, Sichuan, China; 2 Department of Otolaryngology, Dazhou Central Hospital, Dazhou, Sichuan, China; Indiana University School of Medicine, UNITED STATES OF AMERICA

## Abstract

**Background:**

Respiratory infections and tuberculosis (RIT) remain major contributors to global morbidity and mortality. In rapidly ageing societies, demographic shifts may decouple disease occurrence from fatal outcomes. Japan offers a representative setting to examine long-term spatiotemporal patterns of RIT burden in an advanced ageing context.

**Methods:**

A population-based spatiotemporal analysis of Japan from 2010 to 2023 was conducted using standardized national and prefectural estimates. Annual RIT prevalence and deaths were examined by sex and age group. Long-term trends were quantified using estimated annual percentage change (EAPC) from log-linear models of rates, and changes in the etiological composition of RIT mortality were assessed by age and sex.

**Results:**

National RIT prevalence fell from 31.40 million cases in 2010 to 27.52 million in 2023, and the prevalence rate decreased from 24,536.02 to 22,070.66 per 100,000 (EAPC −1.11). In 2023, prevalent cases were similar between females (13.95 million) and males (13.57 million). Adults aged 75 years and older increased from 2.47 million prevalent cases in 2010 to 3.28 million in 2023 despite declining rates. Total RIT deaths decreased from 104,048 in 2010–98,091 in 2023, while the death rate changed from 81.31 to 78.68 per 100,000 (EAPC 0.83). Mortality remained higher in males than females, with 61,683 versus 36,407 deaths in 2023, and was concentrated in adults aged 75 years and older (87,545 deaths), although their death rate declined from 629.40 to 429.91 per 100,000. Across prefectures, prevalence rates declined universally (−8.47% to −56.30%), whereas deaths increased in most prefectures (−10.74% to 153.64%), with the largest rises observed in Saitama (153.64%), Chiba (130.41%), and Osaka (63.79%). Etiology-specific patterns shifted, with Streptococcus pneumoniae declining from 28.9% to 20.4% of deaths, while Staphylococcus aureus rose from 12.2% to 19.0% and Legionella spp. from 8.6% to 11.5%, most notably among adults aged 75 years and older.

**Conclusions:**

In ageing settings exemplified by Japan, declining RIT prevalence can coexist with persistent, older-adult–concentrated mortality, subnational inequality, and an evolving etiological profile. Age-focused care, prefecture-tailored prevention, and etiology-aware management may reduce avoidable deaths and geographic disparities.

## Introduction

Respiratory infections and tuberculosis (RIT) remain a major cause of morbidity and mortality worldwide, imposing a substantial and persistent burden on health systems despite long-term improvements in prevention and treatment [1]. Lower respiratory tract infections continue to rank among the leading causes of death globally, while tuberculosis remains a critical public health challenge, particularly among vulnerable populations [2,3]. In high-income countries, demographic transitions characterized by rapid population ageing have reshaped the epidemiology of respiratory infections, with older adults experiencing disproportionately higher risks of severe disease and death [4,5]. Japan, as one of the most rapidly ageing societies in the world, provides a unique and important context for examining long-term patterns in the burden of RIT and their implications for public health planning.

Previous studies on respiratory infections and tuberculosis in Japan have primarily focused on national-level estimates or specific pathogens, often overlooking subnational heterogeneity and long-term temporal dynamics [6–10]. National averages may mask substantial differences across prefectures, where variations in population structure, healthcare accessibility, urbanization, and local public health capacity can lead to markedly different disease burdens and trends [9,11]. Moreover, many studies have examined either prevalence or mortality in isolation, without jointly assessing how patterns of disease occurrence relate to fatal outcomes across age and sex groups [12]. Within the GBD framework, RIT represents a standardized composite category integrating multiple major respiratory infectious conditions across populations and demographic groups. Although these conditions differ biologically and epidemiologically, evaluating them collectively may better reflect the overall healthcare and mortality burden imposed by respiratory infectious diseases in rapidly ageing societies such as Japan. A comprehensive spatiotemporal analysis that integrates prevalence, mortality, age structure, and geographic variation is therefore essential to fully characterize the burden of RIT and to identify populations and regions at highest risk.

In this study, we conducted a population-based spatiotemporal analysis of the burden of respiratory infections and tuberculosis in Japan and its prefectures from 2010 to 2023. Using standardized estimates, we systematically assessed trends in prevalence and mortality by sex and age group, quantified geographic disparities at the prefectural level, and examined temporal changes in disease burden using both absolute and relative metrics. By integrating national trends with subnational patterns, this study aims to provide a comprehensive and up-to-date overview of the evolving epidemiology of RIT in Japan, offering evidence to support targeted public health interventions and resource allocation in an ageing society.

## Materials and methods

### Data sources

Data on RIT were extracted specifically for Japan from the Global Burden of Disease (GBD) study, which provides standardized estimates of disease burden across locations, age groups, sex, and calendar years [13]. For Japan, the GBD study offers nationally representative and internally consistent estimates at both the national level and the prefectural level, enabling detailed subnational analyses [14]. We extracted annual estimates of prevalence and mortality (deaths) for Japan, including absolute numbers and rates per 100,000 population, stratified by sex and age group. These data allowed for a comprehensive assessment of temporal trends and demographic patterns in RIT burden across the Japanese population.

### Case definition and measures

RIT was defined as a composite cause category comprising tuberculosis, lower respiratory infections, upper respiratory infections, otitis media, and COVID-19, based on standard disease classification mapping to International Classification of Diseases (ICD) codes [15,16]. Specifically, the corresponding ICD-10 codes included A10–A14, A15–A18.89, A19–A19.9, A48.1, A70, B90–B90.9, B96.0–B96.1, B97.21, B97.4–B97.6, H65, H70.93, J00–J06.9, J09–J18.2, J18.8–J18.9, J19.6–J22.9, J36–J36.0, J85.1, J91.0, K67.3, K93.0, M49.0, N74.0–N74.1, P23–P23.9, P37.0, U04–U04.9, and U84.3, while ICD-9 codes included 010–019.9, 079.82, 137–137.9, 320.4, 381–383.9, 460–469, 470.0, 475–475.9, 480–484, 484.1–490.9, 510–511.9, 513.0–513.9, 730.4–730.6, 770.0, V01.1, V01.82, V03.2, V04.7, V04.81, V12.01, V12.61, and V74.1. We assessed two primary burden measures: prevalence, defined as the total number of existing RIT cases in a given year, and mortality (deaths), defined as the number of deaths attributable to RIT; for each measure, both absolute numbers and rates per 100,000 population were analyzed to provide complementary perspectives on disease burden.

### Study population and stratification

Analyses were conducted for Japan overall and all 47 prefectures. All estimates were stratified by sex (male, female, and both sexes combined) and age group, including all ages, 0–14 years, 15–49 years, 50–74 years, and ≥75 years. These age categories were selected to reflect major life stages and to capture the disproportionate contribution of older adults to RIT-related mortality.

### Temporal trend analysis

Temporal trends in RIT prevalence and mortality were evaluated using descriptive and quantitative approaches. Annual changes in absolute numbers and rates were first visualized at the national level by sex and age group. To quantify long-term trends at the prefectural level, the estimated annual percentage change (EAPC) in prevalence and death rates from 2010 to 2023 was calculated using a log-linear regression model [17]. Specifically, the natural logarithm of the rate was modeled as: ln(Rate) = alpha + beta × year + error, where Rate represents the rate per 100,000 population and year denotes calendar year. EAPC was derived from the regression coefficient using the following formula: EAPC = 100 × (exp(beta) − 1). EAPC estimates with 95% confidence intervals crossing zero were interpreted as indicating no clear long-term monotonic trend. When applicable, 95% confidence intervals (CIs) for EAPC were calculated as: 95% CI = 100 × (exp(beta ± 1.96 × SE) − 1), where SE denotes the standard error of the estimated beta coefficient.

### Etiology-specific analysis

To explore changes in the pathogen composition of RIT, etiology-specific analyses were conducted. Death rates attributable to major etiological categories were examined over time and stratified by age group. In addition, the distribution of RIT cases by etiology was compared between 2010 and 2023, stratified by sex and age group, and presented as both absolute numbers and relative contributions (%). These analyses were performed to characterize shifts in the etiological structure of RIT and to assess heterogeneity across demographic subgroups [10,15,18]. Etiological categories in the GBD framework represent modeled cause-specific attributions rather than laboratory-confirmed infections. Causes were treated as mutually exclusive within the GBD estimation framework, and deaths involving multiple potential infectious contributors were assigned according to probabilistic cause attribution models used by the GBD study.

### Statistical analysis

Temporal and prefecture-level trend metrics were summarized for 2010–2023. Prefecture-specific estimated annual percentage change (EAPC) values for rates were calculated using a log-linear regression of the natural logarithm of the rate on calendar year, with EAPC derived from the regression coefficient. Prefecture-level burden in 2023 and spatial heterogeneity in temporal trends were visualized using choropleth maps for rates and EAPCs, respectively. To complement EAPC, percentage changes in absolute numbers between 2010 and 2023 were additionally computed for each prefecture and reported in supplementary analyses. All data management, analyses, visualizations, and map generation were performed in R (version 4.2.2; R Foundation for Statistical Computing, Vienna, Austria). Prefecture-level maps were generated using publicly available administrative boundary data for Japan obtained through the geodata package (GADM level-1 boundaries), without the use of proprietary basemaps or satellite imagery. Given the descriptive nature of the study, no formal hypothesis testing was conducted [19].

### Ethics statement

This study was based exclusively on publicly available, aggregated, and anonymized national and prefectural-level data. No individual-level data were collected, and no human participants were directly involved. The study protocol was reviewed and approved by the Ethics Committee of Dazhou Central Hospital (approval number: 202512−021). Informed consent was waived in accordance with institutional and national research guidelines.

## Results

### National trends in prevalence of respiratory infections and tuberculosis in Japan

**Fig 1:**
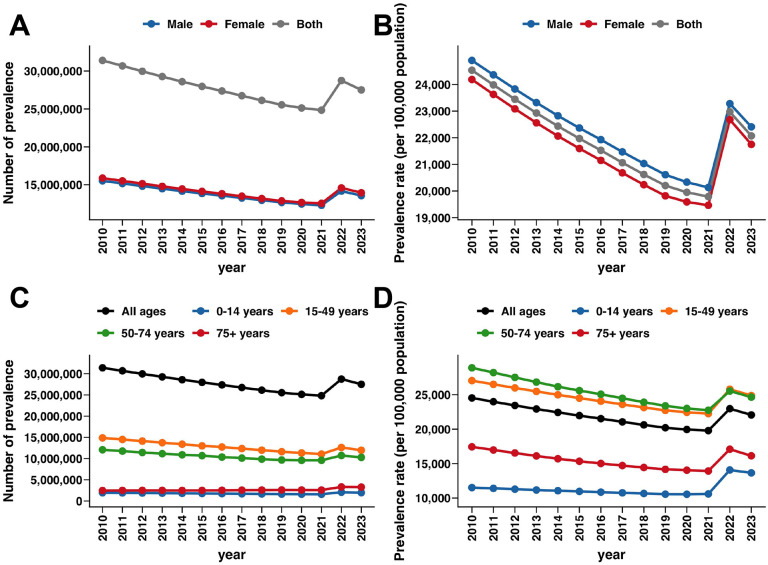
Temporal trends in the prevalence of respiratory infections and tuberculosis (RIT) in Japan from 2010 to 2023, stratified by sex and age group. (A) Trends in the number of prevalent cases by sex (male, female, and both sexes). (B) Trends in prevalence rates (per 100,000 population) by sex. (C) Trends in the number of prevalent cases across age groups (all ages, 0–14 years, 15–49 years, 50–74 years, and ≥75 years). (D) Trends in prevalence rates (per 100,000 population) across age groups.

At the national level, the prevalence of RIT in Japan declined overall from 2010 to 2021, followed by a marked rebound in 2022 and a slight decrease in 2023 (Fig 1A–1D). Prevalent cases decreased from 31.40 million (95% UI: 27.76–35.36 million) in 2010 to 27.52 million (95% UI: 24.39–31.68 million) in 2023 (Table 1). In parallel, the prevalence rate declined from 24,536.02 per 100,000 population (95% UI: 21,692.97–27,632.28) to 22,070.66 per 100,000 population (95% UI: 19,565.94–25,413.43), with an EAPC of −1.11 (95% CI: −1.79 to −0.43) over 2010–2023 (Table 1). Sex-specific trends were highly consistent, with males and females showing similar trajectories in prevalence numbers (Fig 1A) and prevalence rates (Fig 1B); in 2023, prevalent cases were 13.95 million (95% UI: 12.37–16.11 million) in females and 13.57 million (95% UI: 12.02–15.63 million) in males, and the corresponding EAPCs were comparable (Table 1).

**Table 1:** Prevalence and temporal trends of respiratory infections and tuberculosis in Japan by sex, age group, and prefecture, 2010–2023.

Feature	Prevalence, Cases in 2010	Prevalence rate in 2010	Prevalence, Cases in 2023	Prevalence rate in 2023	% change	EAPC (95% CI)
**Japan**	31397531 (27759420–35359659)	24536.02 (21692.97 to 27632.28)	27516088 (24393381–31683613)	22070.66 (19565.94 to 25413.43)	−38.21 (−42.87 to −30.65)	−1.11 (−1.79 to −0.43)
**Sex group**						
Female	15878622 (14045805–17872421)	24187.25 (21395.39 to 27224.32)	13946353 (12370475–16114464)	21750.26 (19292.58 to 25131.58)	−38.31 (−42.84 to −30.44)	−1.12 (−1.81 to −0.41)
Male	15518909 (13723003–17510077)	24903.45 (22021.53 to 28098.71)	13569735 (12015310–15633770)	22409.94 (19842.86 to 25818.62)	−38.11 (−43.03 to −30.76)	−1.11 (−1.76 to −0.45)
**Age group**						
All ages	31397531 (27759420–35359659)	24536.02 (21692.97 to 27632.28)	27516088 (24393381–31683613)	22070.66 (19565.94 to 25413.43)	−38.21 (−42.87 to −30.65)	−1.11 (−1.79 to −0.43)
0-14 years	1952786 (1682268–2206731)	11517.33 (9921.84 to 13015.07)	1978130 (1696916–2347505)	13651.06 (11710.4 to 16200.11)	−43.28 (−50.55 to −33.6)	0.71 (−0.45 to 1.88)
15-49 years	14886215 (12469493–17917191)	27050.3 (22658.79 to 32558.01)	11963874 (10220316–14380104)	24886.58 (21259.73 to 29912.69)	−52.85 (−56.85 to −45.43)	−0.95 (−1.6 to −0.29)
50-74 years	12088102 (9669676–14661139)	28912.41 (23128 to 35066.62)	10289876 (8465605–12106855)	24649.4 (20279.36 to 29001.97)	−25 (−31.15 to −17.34)	−1.47 (−2.03 to −0.91)
75 + years	2470428 (1980512–3035547)	17435.63 (13977.93 to 21424.09)	3284209 (2758943–3917545)	16127.92 (13548.47 to 19238.08)	68.22 (48.65 to 95.82)	−0.91 (−1.81 to −0.01)
**Prefectures**						
Aichi	1926456 (1703478–2194573)	26024.51 (23012.3 to 29646.51)	1736414 (1537317–2008019)	23129.5 (20477.47 to 26747.35)	−30.26 (−36.32 to −21.09)	−1.21 (−1.94 to −0.48)
Akita	270543 (239944–305221)	24906.32 (22089.38 to 28098.85)	197451 (175998–224055)	21462.98 (19131.03 to 24354.83)	−56.3 (−59.57 to −51.62)	−1.32 (−1.78 to −0.86)
Aomori	355342 (314344–403295)	25812.5 (22834.36 to 29295.89)	258590 (230228–291282)	21543.49 (19180.66 to 24267.12)	−53.94 (−57.84 to −48.99)	−1.51 (−2.01 to −1)
Chiba	1532661 (1361378–1737295)	24703.6 (21942.84 to 28001.91)	1391769 (1237816–1608352)	22218.36 (19760.64 to 25675.91)	−29.11 (−34.97 to −20.41)	−1.12 (−1.79 to −0.44)
Ehime	351316 (311911–399596)	24566.26 (21810.84 to 27942.32)	284903 (252136–329494)	21947.62 (19423.35 to 25382.67)	−48.01 (−52.18 to −41.29)	−1.17 (−1.8 to −0.54)
Fukui	189984 (169001–215322)	23568.04 (20965.07 to 26711.28)	167665 (147358–197254)	22259.49 (19563.55 to 26187.71)	−41.86 (−46.76 to −33.17)	−0.91 (−1.61 to −0.21)
Fukuoka	1225060 (1097689–1392148)	24171.49 (21658.35 to 27468.27)	1128316 (994598–1314608)	22083.36 (19466.24 to 25729.46)	−34.8 (−40.49 to −25.07)	−1.02 (−1.75 to −0.29)
Fukushima	499906 (442095–561301)	24657.78 (21806.25 to 27686.07)	389490 (348145–442370)	21827.15 (19510.16 to 24790.57)	−49.05 (−53.07 to −43.72)	−1.16 (−1.72 to −0.6)
Gifu	499108 (442172–565999)	24003.92 (21265.68 to 27220.96)	418977 (370741–480491)	21672.98 (19177.78 to 24854.96)	−42.3 (−46.89 to −35.24)	−1.07 (−1.74 to −0.39)
Gunma	495749 (437858–562552)	24699.98 (21815.63 to 28028.33)	442771 (387899–522911)	23223.37 (20345.33 to 27426.69)	−36.94 (−42.73 to −27.03)	−0.97 (−1.72 to −0.21)
Hiroshima	683813 (608066–780540)	23923.09 (21273.11 to 27307.09)	611437 (540066–712282)	22197.28 (19606.26 to 25858.3)	−39.17 (−44.52 to −30.31)	−0.97 (−1.73 to −0.21)
Hokkaidō	1362083 (1201963–1548667)	24734.7 (21827.01 to 28122.96)	1092044 (977100–1238951)	21447.63 (19190.14 to 24332.87)	−47.04 (−51.23 to −41.73)	−1.29 (−1.85 to −0.72)
Hyōgo	1361540 (1201170–1558687)	24381.47 (21509.67 to 27911.83)	1134708 (1008112–1282492)	21122.56 (18765.99 to 23873.57)	−42.08 (−46.71 to −36.15)	−1.26 (−1.85 to −0.68)
Ibaraki	738156 (652919–847165)	24887.24 (22013.44 to 28562.52)	664516 (582226–781091)	23601.97 (20679.25 to 27742.46)	−35.78 (−41.81 to −24.71)	−0.95 (−1.73 to −0.17)
Ishikawa	279682 (246640–318049)	23917.32 (21091.71 to 27198.31)	249064 (220396–288295)	22307.94 (19740.24 to 25821.71)	−38.92 (−43.93 to −31.15)	−0.98 (−1.66 to −0.29)
Iwate	331678 (294322–375830)	24941.71 (22132.58 to 28261.9)	253522 (225762–287108)	21786.94 (19401.34 to 24673.19)	−51.38 (−55.24 to −45.83)	−1.26 (−1.87 to −0.65)
Kagawa	241627 (213687–272339)	24282.22 (21474.42 to 27368.58)	198467 (176676–226489)	21324.51 (18983.16 to 24335.39)	−45.27 (−49.44 to −38.9)	−1.25 (−1.84 to −0.66)
Kagoshima	425937 (377313–482021)	24989.23 (22136.47 to 28279.59)	347180 (306424–398787)	22439.45 (19805.26 to 25774.98)	−48.2 (−52.67 to −41.24)	−1.22 (−1.89 to −0.55)
Kanagawa	2179840 (1927426–2462194)	24113.6 (21321.37 to 27237.04)	2014185 (1780655–2332372)	21848.84 (19315.63 to 25300.37)	−28.78 (−34.69 to −19.43)	−1.03 (−1.73 to −0.31)
Kumamoto	429575 (381362–483071)	23649.73 (20995.43 to 26594.87)	381244 (335372–445795)	22393.31 (19698.9 to 26184.9)	−41.74 (−46.66 to −33.78)	−0.92 (−1.65 to −0.17)
Kyōto	628212 (557559–709595)	23833.57 (21153.06 to 26921.13)	551751 (487628–631302)	21731.91 (19206.26 to 24865.18)	−40.5 (−45.16 to −32.68)	−1.05 (−1.75 to −0.35)
Kōchi	191501 (168420–215931)	25065.16 (22044.2 to 28262.83)	140751 (125938–159801)	21084.45 (18865.53 to 23938.15)	−54.04 (−57.84 to −49.34)	−1.49 (−2.02 to −0.96)
Mie	451683 (402342–512516)	24357.79 (21697.01 to 27638.32)	386271 (339661–449713)	22291.99 (19602.11 to 25953.3)	−39.79 (−44.63 to −30.8)	−1.07 (−1.78 to −0.35)
Miyagi	563865 (500896–636722)	24008.22 (21327.12 to 27110.29)	507423 (447523–591013)	22413.14 (19767.32 to 26105.34)	−35.83 (−41.39 to −27.18)	−0.95 (−1.66 to −0.24)
Miyazaki	274615 (241155–309038)	24218.91 (21267.97 to 27254.77)	236420 (206779–276795)	22641.2 (19802.56 to 26507.76)	−44.11 (−48.95 to −36.5)	−0.99 (−1.71 to −0.27)
Nagano	497613 (442038–562699)	23127.17 (20544.24 to 26152.11)	435595 (385688–500949)	21705.22 (19218.38 to 24961.73)	−40.76 (−45.72 to −32.33)	−0.91 (−1.59 to −0.22)
Nagasaki	348707 (309832–395042)	24442.5 (21717.54 to 27690.3)	276931 (244889–319548)	21808.11 (19284.87 to 25164.18)	−51.38 (−55.53 to −45.8)	−1.18 (−1.79 to −0.57)
Nara	334095 (298522–380459)	23873.68 (21331.72 to 27186.77)	283753 (249951–328914)	21962.77 (19346.42 to 25458.29)	−41.42 (−46.48 to −33)	−1.06 (−1.75 to −0.35)
Niigata	559293 (497554–637168)	23563.78 (20962.64 to 26844.76)	454555 (401688–523416)	21353.8 (18870.23 to 24588.7)	−46.12 (−50.18 to −40.21)	−1.04 (−1.65 to −0.43)
Okayama	465097 (407182–531660)	23928.25 (20948.64 to 27352.77)	408885 (360083–477282)	22027.64 (19398.56 to 25712.31)	−40.3 (−45.04 to −31.79)	−1.04 (−1.77 to −0.3)
Okinawa	354699 (315608–404743)	25444 (22639.85 to 29033.88)	393066 (338634–488369)	26616.67 (22930.81 to 33070.2)	−8.47 (−18.35 to 10.23)	−0.5 (−1.59 to 0.59)
Saga	204231 (181102–229378)	24004.98 (21286.42 to 26960.71)	180585 (159250–212709)	22649.2 (19973.36 to 26678.26)	−42.69 (−47.77 to −34.15)	−0.98 (−1.7 to −0.26)
Saitama	1775366 (1587806–2014570)	24691.92 (22083.32 to 28018.78)	1607537 (1428838–1841353)	21969.57 (19527.37 to 25165.04)	−30.23 (−36 to −21.11)	−1.14 (−1.82 to −0.46)
Shiga	331426 (293542–372030)	23511 (20823.58 to 26391.43)	332861 (288786–399368)	23703.74 (20565.05 to 28439.81)	−21.83 (−30.01 to −7.11)	−0.66 (−1.6 to 0.3)
Shimane	169079 (149904–191110)	23576.91 (20903.13 to 26649)	132783 (118594–150301)	20319.36 (18147.99 to 23000.05)	−51.96 (−55.47 to −47.38)	−1.29 (−1.79 to −0.79)
Shizuoka	905931 (804247–1018959)	24072.66 (21370.67 to 27076.06)	775438 (684956–888521)	21736.16 (19199.86 to 24905.95)	−40.15 (−45.34 to −32.77)	−1.06 (−1.71 to −0.41)
Tochigi	502418 (444355–575913)	25042.66 (22148.53 to 28705.96)	433395 (382645–502711)	22869.02 (20191.07 to 26526.65)	−38.55 (−44.05 to −29.5)	−1.06 (−1.76 to −0.34)
Tokushima	196667 (173567–224070)	25052.3 (22109.68 to 28542.99)	147930 (132641–167271)	21289.08 (19088.86 to 24072.56)	−51.71 (−55.56 to −47.48)	−1.4 (−1.88 to −0.92)
Tottori	143153 (126765–161756)	24296.92 (21515.41 to 27454.23)	122165 (107031–143804)	22649.61 (19843.74 to 26661.47)	−44.34 (−49.15 to −35.07)	−1.05 (−1.75 to −0.36)
Toyama	262497 (232905–297514)	23999.68 (21294.09 to 27201.16)	214029 (190968–245339)	21086.3 (18814.36 to 24171.02)	−46.13 (−50.04 to −40.59)	−1.24 (−1.8 to −0.67)
Tōkyō	3237037 (2855538–3703043)	24638.34 (21734.6 to 28185.29)	3096246 (2764687–3555891)	21725.26 (19398.83 to 24950.43)	−27.63 (−33.67 to −19.25)	−1.13 (−1.81 to −0.44)
Wakayama	245635 (219332–280799)	24528.59 (21902.01 to 28039.97)	197353 (174379–225711)	22052.53 (19485.38 to 25221.27)	−49.55 (−53.89 to −43.21)	−1.14 (−1.74 to −0.53)
Yamagata	277567 (243836–310990)	23700.16 (20819.99 to 26553.97)	226309 (200808–259836)	21891.26 (19424.57 to 25134.41)	−48.63 (−52.71 to −41.53)	−1.01 (−1.63 to −0.39)
Yamaguchi	353833 (315276–404422)	24396.88 (21738.37 to 27884.96)	274733 (242706–311785)	21150.41 (18684.79 to 24002.92)	−51.82 (−55.65 to −47.44)	−1.29 (−1.86 to −0.71)
Yamanashi	207343 (184220–232081)	24027.06 (21347.6 to 26893.72)	170395 (152194–194537)	21492.59 (19196.75 to 24537.7)	−43.88 (−48.22 to −37.45)	−1.14 (−1.73 to −0.55)
Ōita	284853 (249863–319929)	23840.89 (20912.37 to 26776.59)	233196 (206873–268890)	21373.97 (18961.3 to 24645.56)	−47.73 (−51.7 to −40.88)	−1.12 (−1.75 to −0.49)
Ōsaka	2251060 (1979720–2559981)	25415.33 (22351.8 to 28903.17)	1933017 (1718214–2212914)	22071.35 (19618.71 to 25267.23)	−41.2 (−46.44 to −34.95)	−1.3 (−1.96 to −0.63)

Data in parentheses are 95% uncertainty intervals for cases and rates, and 95% confidence intervals for EAPC.

Age-stratified analyses showed substantial heterogeneity in both prevalence numbers (Fig 1C) and prevalence rates (Fig 1D). Individuals aged 15–49 years and 50–74 years consistently contributed the largest numbers of prevalent cases, and both groups exhibited clear declines in absolute numbers and rates between 2010 and 2023 (Table 1). In contrast, prevalent cases among those aged ≥75 years increased from 2.47 million (95% UI: 1.98–3.04 million) in 2010 to 3.28 million (95% UI: 2.76–3.92 million) in 2023, despite a modest decline in prevalence rate and a negative EAPC (Table 1), indicating a growing contribution of older adults to the national prevalence burden. Prevalence in children aged 0–14 years remained relatively low in absolute numbers (Fig 1C), while the prevalence rate increased from 11,517.33 to 13,651.06 per 100,000 population over 2010–2023 (EAPC 0.71; 95% CI: −0.45 to 1.88) (Table 1; Fig 1D).

### National trends in mortality of respiratory infections and tuberculosis in Japan

**Fig 2:**
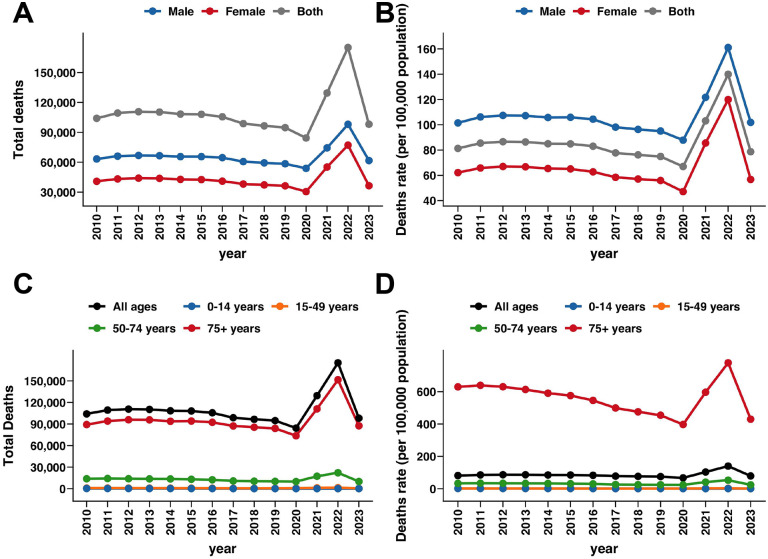
Temporal trends in mortality from respiratory infections and tuberculosis (RIT) in Japan from 2010 to 2023, stratified by sex and age group. (A) Trends in the number of deaths by sex (male, female, and both sexes combined). (B) Trends in death rates (per 100,000 population) by sex. (C) Trends in the number of deaths across age groups (all ages, 0–14 years, 15–49 years, 50–74 years, and ≥75 years). (D) Trends in death rates (per 100,000 population) across age groups.

Building on the observed decline in prevalence at the national level, we further examined mortality patterns of RIT in Japan from 2010 to 2023. Overall mortality remained relatively stable from 2010 to 2019, followed by a pronounced increase in 2021–2022 and a subsequent decline in 2023. In absolute terms, the number of deaths decreased slightly from 104,048 (95% UI: 85,703–118,177) in 2010–98,091 (95% UI: 78,865–114,618) in 2023 (Table 2). Over the same period, the death rate declined modestly from 81.31 per 100,000 population (95% UI: 66.97–92.35) to 78.68 per 100,000 population (95% UI: 63.26–91.93), with an overall EAPC of 0.83 (95% CI: −1.45 to 3.16), indicating no significant long-term monotonic trend (Table 2). Sex-specific analyses demonstrated consistently higher mortality among males than females across the study period (Fig 2A–2B). In 2023, there were 61,683 deaths (95% UI: 51,320–72,761) among males and 36,407 deaths (95% UI: 24,350–47,882) among females, with corresponding death rates of 101.87 and 56.78 per 100,000 population, respectively (Table 2), while the temporal trajectories for both sexes remained broadly parallel.

**Table 2:** Deaths and temporal trends of respiratory infections and tuberculosis in Japan by sex, age group, and prefecture, 2010–2023.

Feature	Deaths, Cases in 2010	Death rate in 2010	Deaths, Cases in 2023	Death rate in 2023	% change	EAPC (95% CI)
**Japan**	104048 (85703–118177)	81.31 (66.97 to 92.35)	98091 (78865–114618)	78.68 (63.26 to 91.93)	46.49 (23.86 to 71.58)	0.83 (−1.45 to 3.16)
**Sex group**						
Female	40811 (28677–49776)	62.17 (43.68 to 75.82)	36407 (24350–47882)	56.78 (37.98 to 74.68)	41.16 (2.08 to 80.97)	0.87 (−2.04 to 3.87)
Male	63237 (54213–71635)	101.48 (87 to 114.95)	61683 (51320–72761)	101.87 (84.75 to 120.16)	49.84 (28.28 to 78.69)	0.77 (−1.09 to 2.67)
**Age group**						
0-14 years	257 (223–292)	1.51 (1.31 to 1.72)	113 (99–129)	0.78 (0.68 to 0.89)	−81.96 (−84.97 to −78.72)	−6.76 (−8.13 to −5.37)
15-49 years	831 (744–927)	1.51 (1.35 to 1.69)	443 (397–493)	0.92 (0.83 to 1.03)	−69.28 (−73.23 to −64.33)	0.21 (−3.75 to 4.34)
50-74 years	13782 (12206–15405)	32.96 (29.2 to 36.85)	9990 (8563–11628)	23.93 (20.51 to 27.86)	−36.62 (−44.81 to −26.22)	−0.4 (−3.36 to 2.65)
75 + years	89179 (71717–101784)	629.4 (506.16 to 718.36)	87545 (69760–103005)	429.91 (342.57 to 505.83)	78.19 (51.22 to 108.42)	−1.81 (−4.01 to 0.44)
**Prefectures**						
Aichi	4983 (4171–5669)	67.32 (56.35 to 76.58)	4919 (3981–5682)	65.52 (53.02 to 75.68)	82.4 (50.24 to 114.5)	1.12 (−1.24 to 3.53)
Akita	1333 (1126–1520)	122.69 (103.66 to 139.97)	1077 (865–1283)	117.04 (94.02 to 139.48)	40.61 (16.31 to 71.77)	0.76 (−1.31 to 2.87)
Aomori	1469 (1203–1681)	106.72 (87.41 to 122.09)	1524 (1239–1806)	126.93 (103.25 to 150.44)	64.79 (36.48 to 98.02)	1.35 (−0.51 to 3.25)
Chiba	4342 (3563–5043)	69.98 (57.44 to 81.28)	4883 (4005–5716)	77.95 (63.94 to 91.25)	130.41 (91.11 to 172.88)	1.92 (−0.26 to 4.14)
Ehime	1411 (1164–1621)	98.64 (81.39 to 113.33)	1285 (1012–1533)	99 (77.96 to 118.11)	25.76 (3.96 to 50.74)	0.47 (−1.37 to 2.35)
Fukui	815 (665–959)	101.14 (82.46 to 119.02)	667 (531–798)	88.53 (70.54 to 105.99)	31.6 (7.63 to 58.13)	−0.06 (−2.09 to 2.02)
Fukuoka	4176 (3325–4839)	82.4 (65.61 to 95.48)	3849 (2955–4716)	75.33 (57.83 to 92.3)	46.17 (18.93 to 73.46)	0.52 (−1.91 to 3.01)
Fukushima	1889 (1535–2183)	93.19 (75.73 to 107.69)	1627 (1277–1949)	91.18 (71.55 to 109.22)	41.84 (18.07 to 70.73)	0.7 (−1.38 to 2.82)
Gifu	1748 (1428–2002)	84.06 (68.7 to 96.3)	1593 (1274–1882)	82.42 (65.92 to 97.37)	53.59 (28.51 to 82.61)	0.91 (−1.26 to 3.12)
Gunma	1939 (1579–2244)	96.61 (78.68 to 111.83)	1870 (1487–2252)	98.06 (77.97 to 118.1)	57.16 (28.64 to 88.24)	0.73 (−1.35 to 2.85)
Hiroshima	2409 (1967–2786)	84.28 (68.82 to 97.47)	2140 (1705–2584)	77.7 (61.9 to 93.79)	29.73 (7.03 to 53.37)	0.08 (−2.19 to 2.41)
Hokkaidō	4521 (3688–5189)	82.11 (66.98 to 94.24)	4361 (3477–5123)	85.64 (68.29 to 100.61)	34.37 (12.35 to 58.51)	1.72 (−0.85 to 4.36)
Hyōgo	4345 (3548–4958)	77.82 (63.54 to 88.79)	3857 (3086–4542)	71.8 (57.45 to 84.55)	36.11 (15.41 to 61.67)	1.01 (−1.74 to 3.85)
Ibaraki	2562 (2109–2934)	86.37 (71.09 to 98.92)	2607 (2122–3090)	92.6 (75.38 to 109.74)	84.7 (52.44 to 118.49)	1.41 (−0.57 to 3.42)
Ishikawa	1066 (848–1237)	91.18 (72.5 to 105.82)	880 (706–1041)	78.82 (63.21 to 93.27)	13.25 (−6.93 to 36.08)	−0.57 (−2.77 to 1.67)
Iwate	1420 (1167–1632)	106.76 (87.76 to 122.71)	1218 (976–1440)	104.65 (83.87 to 123.75)	26.47 (5.7 to 50.59)	0.45 (−1.57 to 2.5)
Kagawa	1005 (836–1158)	100.99 (84.04 to 116.36)	805 (647–953)	86.52 (69.53 to 102.36)	10.68 (−9.42 to 29.85)	−0.18 (−2.74 to 2.44)
Kagoshima	1958 (1560–2281)	114.89 (91.5 to 133.8)	1744 (1361–2091)	112.71 (87.94 to 135.15)	19.98 (−0.27 to 44.44)	0.32 (−1.56 to 2.24)
Kanagawa	5415 (4483–6227)	59.91 (49.59 to 68.88)	5269 (4207–6238)	57.16 (45.63 to 67.67)	64.05 (35.42 to 91.22)	1.16 (−1.45 to 3.84)
Kumamoto	1793 (1432–2088)	98.69 (78.84 to 114.95)	1586 (1209–1892)	93.15 (71.03 to 111.1)	32.02 (10.51 to 57.59)	0.48 (−1.75 to 2.76)
Kyōto	2078 (1684–2382)	78.85 (63.87 to 90.39)	1657 (1328–1947)	65.25 (52.32 to 76.69)	2.95 (−13.24 to 22.66)	−0.27 (−2.98 to 2.52)
Kōchi	923 (750–1070)	120.76 (98.17 to 140.03)	801 (634–989)	119.94 (94.91 to 148.15)	11.89 (−5.37 to 38.33)	0.82 (−1.36 to 3.05)
Mie	1632 (1332–1873)	87.99 (71.86 to 101.03)	1398 (1124–1657)	80.7 (64.89 to 95.64)	48.86 (24.28 to 76.06)	0.39 (−1.77 to 2.59)
Miyagi	1792 (1460–2076)	76.31 (62.18 to 88.4)	1421 (1119–1694)	62.78 (49.41 to 74.84)	47.1 (21.99 to 73.93)	−0.33 (−2.82 to 2.23)
Miyazaki	1116 (889–1287)	98.46 (78.4 to 113.54)	1187 (925–1416)	113.63 (88.56 to 135.58)	65.29 (39.55 to 94.71)	1.59 (−0.54 to 3.78)
Nagano	1749 (1377–2027)	81.27 (64 to 94.2)	1463 (1125–1769)	72.91 (56.04 to 88.14)	19.33 (−1.08 to 45.33)	0.13 (−2.29 to 2.62)
Nagasaki	1572 (1259–1815)	110.17 (88.23 to 127.22)	1363 (1095–1621)	107.32 (86.23 to 127.66)	24.39 (2.03 to 49.87)	0.37 (−1.46 to 2.22)
Nara	1216 (992–1402)	86.9 (70.86 to 100.16)	1141 (895–1362)	88.28 (69.3 to 105.41)	71.44 (42.76 to 104.89)	1.18 (−1.07 to 3.49)
Niigata	2168 (1780–2509)	91.34 (75.01 to 105.71)	1798 (1416–2131)	84.46 (66.54 to 100.12)	19.47 (0.85 to 41.43)	0.02 (−2.01 to 2.09)
Okayama	1959 (1567–2260)	100.79 (80.6 to 116.25)	1695 (1323–1987)	91.29 (71.28 to 107.02)	26.84 (7.01 to 48.19)	−0.3 (−2.47 to 1.91)
Okinawa	811 (664–940)	58.15 (47.6 to 67.46)	780 (612–934)	52.84 (41.46 to 63.22)	32.5 (10.32 to 56.86)	1.24 (−2.25 to 4.86)
Saga	928 (754–1082)	109.03 (88.57 to 127.19)	812 (629–993)	101.87 (78.88 to 124.6)	36.9 (12.7 to 65.56)	0.09 (−1.94 to 2.17)
Saitama	4768 (4021–5462)	66.32 (55.92 to 75.97)	6150 (4921–7221)	84.05 (67.26 to 98.69)	153.64 (111.23 to 201.28)	2.74 (0.55 to 4.98)
Shiga	946 (765–1087)	67.09 (54.25 to 77.13)	788 (619–933)	56.12 (44.05 to 66.46)	29.77 (5.92 to 54.46)	−0.18 (−2.72 to 2.42)
Shimane	775 (625–899)	108.07 (87.14 to 125.36)	531 (406–624)	81.2 (62.07 to 95.43)	−10.74 (−25.77 to 6.52)	−1.5 (−3.74 to 0.78)
Shizuoka	2857 (2306–3263)	75.92 (61.28 to 86.69)	2710 (2177–3201)	75.95 (61.03 to 89.72)	45.83 (22.11 to 75.56)	0.97 (−1.26 to 3.26)
Tochigi	1729 (1435–1993)	86.17 (71.51 to 99.34)	1541 (1215–1801)	81.32 (64.13 to 95.05)	41.6 (18.68 to 67.71)	0.39 (−1.98 to 2.81)
Tokushima	893 (725–1015)	113.7 (92.3 to 129.25)	825 (646–981)	118.74 (93.03 to 141.21)	31.69 (8.77 to 57.43)	0.65 (−1.16 to 2.5)
Tottori	540 (435–635)	91.7 (73.78 to 107.71)	424 (327–515)	78.53 (60.58 to 95.48)	15.53 (−6.5 to 38.16)	−0.13 (−2.18 to 1.97)
Toyama	1106 (899–1276)	101.09 (82.18 to 116.63)	948 (742–1142)	93.41 (73.07 to 112.55)	10.98 (−8.21 to 30.65)	−0.24 (−2.25 to 1.81)
Tōkyō	8472 (7033–9737)	64.48 (53.53 to 74.11)	7805 (6224–9257)	54.77 (43.67 to 64.96)	25.21 (6.15 to 46.88)	0.5 (−2.05 to 3.12)
Wakayama	1099 (889–1282)	109.76 (88.79 to 128.07)	965 (768–1141)	107.78 (85.79 to 127.48)	50.63 (23.78 to 76.71)	0.47 (−1.48 to 2.45)
Yamagata	1284 (1038–1485)	109.64 (88.59 to 126.78)	971 (761–1163)	93.96 (73.61 to 112.46)	22.36 (0.51 to 45.45)	−0.39 (−2.57 to 1.84)
Yamaguchi	1780 (1449–2029)	122.7 (99.89 to 139.89)	1597 (1264–1903)	122.96 (97.27 to 146.51)	23.02 (2.82 to 44.33)	0.3 (−1.41 to 2.03)
Yamanashi	750 (598–875)	86.91 (69.3 to 101.38)	645 (500–775)	81.38 (63.11 to 97.76)	27.45 (5.23 to 53.1)	0.22 (−2 to 2.48)
Ōita	1181 (940–1357)	98.82 (78.64 to 113.57)	1090 (867–1277)	99.91 (79.48 to 117.04)	33.96 (8.11 to 58.79)	0.59 (−1.58 to 2.81)
Ōsaka	7326 (6150–8301)	82.72 (69.43 to 93.73)	7826 (6405–9096)	89.36 (73.13 to 103.86)	63.79 (37.07 to 92.97)	2.04 (−0.25 to 4.38)

Data in parentheses are 95% uncertainty intervals for cases and rates, and 95% confidence intervals for EAPC.

Marked age-specific differences in mortality were observed (Fig 2C–2D). Individuals aged ≥75 years accounted for the vast majority of RIT-related deaths throughout the study period, contributing 89,179 deaths (95% UI: 71,717–101,784) in 2010 and 87,545 deaths (95% UI: 69,760–103,005) in 2023 (Table 2). Although the absolute number of deaths in this age group remained high, the death rate decreased from 629.40 to 429.91 per 100,000 population, while the corresponding EAPC (−1.81; 95% CI: −4.01 to 0.44) indicated no clear long-term monotonic trend (Table 2; Fig 2D). In contrast, marked reductions were observed among younger age groups, particularly children aged 0–14 years, in whom deaths decreased by more than 80% and death rates declined from 1.51 to 0.78 per 100,000 population over 2010–2023 (Table 2; Fig 2C–2D). Adults aged 15–49 years and 50–74 years also experienced substantial declines in both deaths and death rates, highlighting a strong age gradient in RIT mortality burden.

### Percentage changes in prevalence and deaths across prefectures

Although RIT prevalence declined nationally, the magnitude of change varied substantially across prefectures between 2010 and 2023. All prefectures experienced reductions in prevalence rates, with percentage decreases ranging from −8.47% to −56.30%. Larger declines were generally observed in northern and northeastern prefectures, such as Akita, Aomori, and Iwate, whereas smaller reductions were noted in several metropolitan or southern regions, including Okinawa, Tokyo, Kanagawa, and Aichi (S1A Fig). Prefecture-specific EAPCs were consistently negative, indicating declining long-term trends nationwide, but with marked spatial heterogeneity in the pace of decline (Table 1).

Across prefectures, changes in RIT mortality between 2010 and 2023 showed marked spatial heterogeneity (S1B Fig; Table 2). In contrast to the uniform decline observed for prevalence, most prefectures experienced increases in the absolute number of deaths, with percentage changes ranging from −10.74% to 153.64%. The largest increases were observed in several highly populated prefectures, including Saitama (153.64%), Chiba (130.41%), Osaka (63.79%), and Tokyo (25.21%), whereas decreases were limited to a small number of prefectures such as Shimane (−10.74%). Prefecture-specific EAPCs for death rates varied widely, with both positive and negative values observed, indicating divergent long-term mortality trajectories across regions despite a broadly similar national pattern (Table 2).

### Prefecture-level spatial distribution and temporal trends of RIT burden

**Fig 3:**
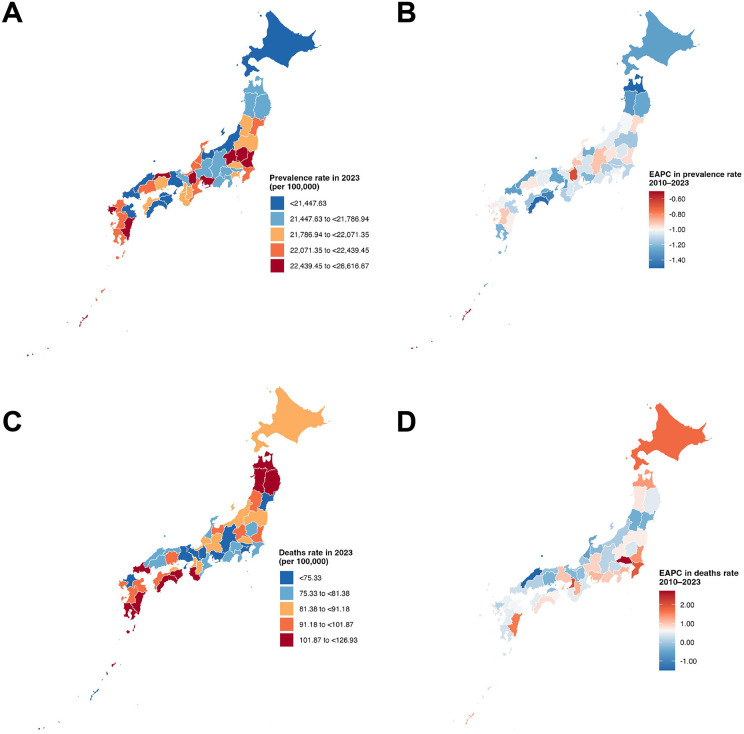
Spatial distribution and temporal trends of prevalence and mortality from respiratory infections and tuberculosis (RIT) across Japanese prefectures. **Maps were generated by the authors in R using publicly available administrative boundary data for Japan.** (A) Prefecture-level prevalence rates (per 100,000 population) in 2023. (B) Estimated annual percentage change (EAPC) in prevalence rates from 2010 to 2023. (C) Prefecture-level death rates (per 100,000 population) in 2023. (D) EAPC in death rates from 2010 to 2023.

In 2023, the spatial distribution of RIT prevalence rates showed clear prefecture-level heterogeneity across Japan (Fig 3A). Prevalence rates ranged from 21,122.56 per 100,000 in Hyōgo to 26,616.67 per 100,000 in Okinawa, with several densely populated prefectures also exhibiting relatively high levels, including Aichi (23,129.50 per 100,000) and Osaka (22,071.35 per 100,000) (Table 1). Despite this cross-sectional variation, temporal trends were broadly downward nationwide: EAPCs in prevalence rates were consistently negative across prefectures, yet the pace of decline differed markedly (Fig 3B; Table 1). The steepest decreases were observed in Aomori (EAPC −1.51), Kōchi (−1.49), and Tokushima (−1.40), whereas declines were comparatively modest in Okinawa (−0.50) and Shiga (−0.66), indicating substantial geographic heterogeneity in long-term improvement (Fig 3B; Table 1).

Building on the observed spatial heterogeneity in disease burden, we further examined RIT mortality at the prefectural level in 2023 and its long-term trends from 2010 to 2023. Marked geographic disparities in mortality rates were evident across Japan in 2023, with higher death rates clustering in several northern prefectures (e.g., parts of the Tohoku region) and selected areas in southwestern Japan, whereas relatively lower rates were observed in the Kanto region and parts of central Japan (Fig 3C). Trend analyses revealed substantial regional variation in the direction and magnitude of change. Although many prefectures showed EAPCs close to zero, indicating broadly stable mortality rates over time, several areas showed positive point estimates of EAPC. In particular, Saitama (EAPC = 2.74, 95% CI 0.55 to 4.98), Osaka (EAPC = 2.04, 95% CI −0.25 to 4.38), and Chiba (EAPC = 1.92, 95% CI −0.26 to 4.14) exhibited relatively high positive EAPC estimates, while declines were observed in a limited number of prefectures, such as Shimane (EAPC = −1.50, 95% CI −3.74 to 0.78) (Fig 3D; Table 2). Overall, these findings indicate persistent and evolving regional inequalities in RIT mortality across Japan, underscoring the need for region-specific strategies to further reduce mortality and address local gaps in prevention and clinical management (Fig 3C–3D; Table 2).

### Etiology-specific patterns of RIT burden by age and sex

**Fig 4:**
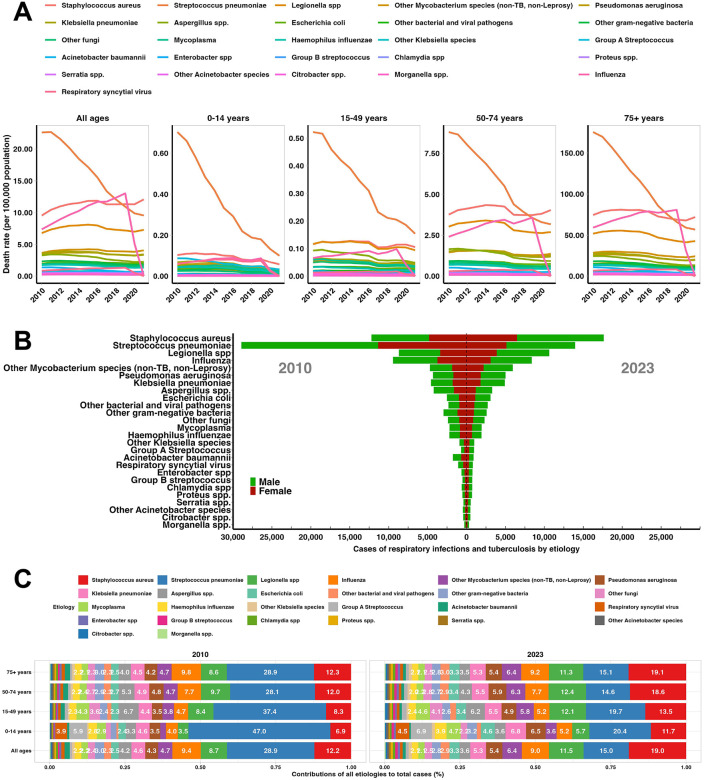
Etiology-specific patterns of respiratory infections and tuberculosis (RIT) in Japan by age group and sex. (A) Trends in death rates (per 100,000 population) attributable to specific etiologies from 2010 to 2020, stratified by age group (all ages, 0–14 years, 15–49 years, 50–74 years, and ≥75 years). (B) Sex-specific distribution of RIT cases by etiology in 2010 and 2023, presented as absolute numbers for males and females. (C) Relative contributions of different etiologies to total RIT cases (%) in 2010 and 2023, stratified by age group.

Across 2010–2023, etiology-specific RIT mortality in Japan showed a marked age-gradient and a clear reshaping of the leading causes over time. The steepest long-term decline was observed for Streptococcus pneumoniae, which decreased consistently in every age stratum, with the most pronounced absolute reductions in adults ≥50 years (Fig 4A). In contrast, several bacterial causes remained comparatively persistent or became more prominent in the later period, especially among older adults, including Staphylococcus aureus and Legionella spp., while influenza fluctuated at intermediate levels rather than falling in parallel with pneumococcal mortality. These temporal patterns translated into a compositional shift between 2010 and 2023 (Fig 4C): the share attributed to S. pneumoniae fell from 28.9% to 20.4% of total deaths (all ages), whereas S. aureus increased from 12.2% to 19.0%, and Legionella spp. increased from 8.6% to 11.5%; influenza remained a major contributor but changed little overall (9.4% to 9.0%) (Fig 4C). The redistribution was most evident in older adults, where S. aureus expanded further (≥75 years: 12.3% → 19.1%) and pneumococcus contracted (≥75 years: 28.9% → 15.1%) (Fig 4C).

In parallel, the absolute burden by etiology remained concentrated in a small number of pathogens and showed persistent sex imbalance (Fig 4B). In both 2010 and 2023, the largest case counts clustered in S. aureus, S. pneumoniae, Legionella spp., influenza, non-tuberculous mycobacteria, and Pseudomonas aeruginosa, with males contributing more cases than females across the leading etiologies (Fig 4B). Age-stratified contribution profiles reinforced this concentration (Fig 4C): in 2010, pneumococcus accounted for an especially large fraction in younger groups (0–14 years: 47.0%; 15–49 years: 37.4%) but these shares declined by 2023 (0–14 years: 20.4%; 15–49 years: 19.7%), accompanied by relative increases in S. aureus (0–14 years: 6.9% → 11.7%; 15–49 years: 8.3% → 13.5%) and Legionella (15–49 years: 8.4% → 12.1%) (Fig 4C). Together, these results indicate that Japan’s RIT mortality burden over the last decade has shifted away from pneumococcal dominance toward S. aureus and Legionella, with a pronounced concentration in older adults and consistently higher burden in males (Fig 4A–4C).

## Discussion

In this population-based spatiotemporal analysis, we provide a comprehensive assessment of the burden of RIT in Japan from 2010 to 2023, integrating prevalence, mortality, age structure, geographic heterogeneity, and etiological composition. Previous global and national studies have consistently identified respiratory infections and tuberculosis as leading contributors to morbidity and mortality, even in high-income settings [20,21]. Our findings extend this literature by demonstrating that, in Japan, long-term declines in RIT prevalence have not translated into proportional reductions in mortality, and that the contemporary burden is increasingly shaped by demographic ageing, regional disparities, and shifts in pathogen composition.

The observed divergence between declining prevalence and relatively stable or fluctuating mortality aligns with recent global burden analyses, which have shown that improvements in infection prevention and treatment may reduce disease occurrence but have a more limited impact on fatal outcomes among high-risk populations [22–25]. Studies from Europe and East Asia have similarly reported stagnation or rebound in respiratory infection mortality during the COVID-19 era, attributed to both direct viral effects and indirect disruptions to healthcare systems [24,26,27]. In Japan, the marked mortality increase during 2021–2022 likely reflects a combination of COVID-19–related deaths, delayed access to care, and increased susceptibility among frail older adults [28]. Our results reinforce emerging evidence that mortality trends in ageing societies are increasingly decoupled from overall infection prevalence, underscoring the need for mortality-focused surveillance alongside traditional incidence-based monitoring.

Age-specific patterns observed in this study are consistent with a growing body of literature highlighting population ageing as a dominant driver of infectious disease mortality in high-income countries. Prior studies have documented substantial declines in respiratory infection mortality among children and younger adults, largely attributable to vaccination, improved nutrition, and healthcare access [26,29,30]. In contrast, mortality among adults aged ≥75 years remains disproportionately high, even when age-specific rates decline [31,32]. Our findings corroborate this demographic paradox: absolute deaths in older adults remained persistently high due to rapid expansion of the oldest age groups. Similar dynamics have been reported for pneumonia, influenza, and sepsis in ageing populations, suggesting that future reductions in RIT mortality will depend less on broad preventive gains and more on optimizing care for older adults with multimorbidity and functional decline.

The pronounced prefectural heterogeneity identified in this study builds on prior evidence that subnational variation plays a critical role in shaping infectious disease outcomes in Japan. Earlier regional studies have shown that differences in population structure, urbanization, healthcare capacity, and long-term care infrastructure contribute to uneven distributions of respiratory disease burden [33–35]. Our analysis extends these findings by quantifying long-term prefecture-specific trends and revealing that several highly populated prefectures experienced both large absolute increases in deaths and positive EAPCs in mortality rates. These patterns suggest that urban density and demographic concentration may amplify mortality burden even in settings with advanced healthcare systems. Importantly, the coexistence of declining prevalence and rising mortality in some prefectures highlights the limitations of relying on national averages and supports calls for region-specific public health strategies.

Etiology-specific analyses further contextualize Japan’s RIT burden within global epidemiological transitions. The sustained decline in Streptococcus pneumoniae–related mortality is consistent with extensive evidence demonstrating the long-term impact of pneumococcal conjugate vaccines and improved clinical management [36,37]. In contrast, the rising contribution of Staphylococcus aureus and Legionella spp., particularly among older adults, mirrors trends reported in other high-income countries, where healthcare-associated infections, environmental exposures, and age-related immune dysfunction play increasingly prominent roles. Previous studies have emphasized the growing importance of non-traditional respiratory pathogens in ageing populations, and our findings provide population-level evidence that this shift is already well underway in Japan. The persistent excess burden among males across major etiologies is also consistent with international research suggesting sex-based differences in immune response, comorbidity profiles, and health-seeking behavior.

Several limitations warrant consideration. Estimates are derived from GBD modeling and therefore reflect integrated data sources and assumptions, which may introduce uncertainty, especially at the prefectural level [38]. Etiology-specific patterns depend on cause-of-death attribution, diagnostic practices, and GBD modeling assumptions, which may vary across regions and over time and may introduce additional uncertainty in prefectural-level etiological estimates. In addition, because RIT was analyzed as a composite category, aggregation of biologically heterogeneous conditions may mask pathogen-specific temporal patterns and epidemiological drivers. Furthermore, inclusion of COVID-19 within the standardized GBD framework likely contributed substantially to the mortality rebound observed after 2020 and may have influenced the observed prevalence–mortality divergence. Despite these constraints, the findings remain robust. Using Japan as a comprehensive representative of an ageing society, this study shows that RIT prevalence continues to decline, whereas mortality remains concentrated in older adults, exhibits pronounced regional disparities, and is increasingly influenced by a changing pathogen landscape. These results underscore the importance of age-focused, region-adapted, and etiology-aware public health strategies that move beyond uniform, population-wide approaches.

In conclusion, using Japan as a comprehensive representative of rapidly ageing societies, we found that RIT prevalence declined nationally from 2010 to 2023, yet mortality remained heavily concentrated in adults aged ≥75 years and varied substantially across prefectures, with several populous regions showing upward mortality trends. The etiological profile of RIT deaths also shifted away from pneumococcal dominance toward a greater contribution of Staphylococcus aureus and Legionella spp., alongside persistent male excess. These findings highlight that, in ageing settings, continued progress will depend on combining broad prevention with targeted, region-specific strategies and pathogen-focused clinical management for older adults to reduce avoidable deaths and narrow geographic inequalities.

## Supporting information

S1 Fig Percentage change in the absolute number of prevalence cases and deaths from respiratory infections and tuberculosis (RIT) across Japanese prefectures between 2010 and 2023. (A) Percentage change in prevalence cases from 2010 to 2023.(B) Percentage change in death cases from 2010 to 2023.Maps were generated by the authors in R using publicly available administrative boundary data for Japan, without the use of Google Maps, satellite imagery, or proprietary basemaps.
(PDF)

S1 Data Dataset. Source data used for prevalence, mortality, temporal trend, and prefectural analyses.
(CSV)

S2 Data Dataset. Source data used for etiology-specific analyses.
(CSV)

## References

[1] da Silva JMN, Diaz-Quijano FA, Sanchez MN, Ramalho WM. Projecting tuberculosis control progress in metropolitan and non-metropolitan areas of Brazil, 2001-2035: a Bayesian age-period-cohort analysis. Infect Dis Poverty. 2025;14(1):125. doi: 10.1186/s40249-025-01400-x 41430721 PMC12720469

[2] Yin L, Yang R, Chen X, Fu Z, He X. Analysis of bacterial pathogen spectrum and epidemiological characteristics of pediatric lower respiratory tract infections: a large sample study based on bronchoalveolar lavage. BMC Infect Dis. 2025;25(1):1794. doi: 10.1186/s12879-025-12361-9 41430111 PMC12752284

[3] Bournot AR, Hart KH, Johnsen S, Givens DI, Lovegrove JA, Ordóñez-Mena JM, et al. Association between serum 25-hydroxyvitamin D status and respiratory tract infections requiring hospital admission: unmatched case-control analysis of ethnic groups from the United Kingdom Biobank cohort. Am J Clin Nutr. 2026;123(2):101179. doi: 10.1016/j.ajcnut.2025.101179 41475552 PMC12917221

[4] Shaaban FL, Groenendijk RW, Baral R, Caballero MT, Crowe JE Jr, Englund JA, et al. The path to equitable respiratory syncytial virus prevention for infants: challenges and opportunities for global implementation. Lancet Glob Health. 2025;13(12):e2165–74. doi: 10.1016/S2214-109X(25)00379-1 41197645

[5] Gustavsson L, Söderlund P, Skovbjerg S, Snygg-Martin U. Clinical presentation and recent health care contacts in patients with invasive group a streptococcal infection, Western Sweden 2020-2024. Infect Dis. 2025;2025:1–11. doi: 10.1080/23744235.2025.2594536 41320631

[6] Krokva D, Mori H, Valenti S, Remez D, Hadano Y, Naito T. Analysis of the impact of crises tuberculosis incidence in Ukraine amid pandemics and war. Sci Rep. 2025;15(1):17045. doi: 10.1038/s41598-025-01723-7 40379776 PMC12084344

[7] Shimokawa Y, Inoue M, Sonoda R, Hanatate C, Watanabe M, Tsuzaki K, et al. Use of Whole-genome Sequencing in a Tuberculosis Outbreak among Young Immigrants in a Japanese Language School, 2024. Int J Mycobacteriol. 2025;14(2):164–9. doi: 10.4103/ijmy.ijmy_46_25 40540661

[8] Seto S, Omori S, Nakamura H, Hijikata M, Keicho N. Single-cell transcriptomic profiling reveals a novel signature of necrotizing granulomatous lesions in the lungs of Mycobacterium tuberculosis-infected C3HeB/FeJ mice. Front Immunol. 2025;16:1624072. doi: 10.3389/fimmu.2025.1624072 40843005 PMC12364640

[9] Moreira-Sousa D, Martins B, Aguiar A, Pinheiro M, Akkerman O, Aksamit TR, et al. Consensus on management of refractory nontuberculous mycobacterial pulmonary disease. Eur Respir J. 2025;2025:2500400. doi: 10.1183/13993003.00400-2025 41198394

[10] GBD 2023 Lower Respiratory Infections and Antimicrobial Resistance Collaborators. Global burden of lower respiratory infections and aetiologies, 1990-2023: a systematic analysis for the Global Burden of Disease Study 2023. Lancet Infect Dis. 2025;2025:S1473-3099(25)00689-9. doi: 10.1016/S1473-3099(25)00689-9 41412141

[11] Nakaya M, Kamishima M, Yamaoka T, Tsuchiya H, Touma T, Saito A, et al. Real-world data on combination treatment with bedaquiline in patients with multidrug-resistant pulmonary tuberculosis in Japan: An interim analysis of post-marketing surveillance. J Infect Chemother. 2025;31(4):102661. doi: 10.1016/j.jiac.2025.102661 39971192

[12] Luo Y, Huang C-C, Howard NC, Wang X, Liu Q, Li X, et al. Paired analysis of host and pathogen genomes identifies determinants of human tuberculosis. Nat Commun. 2024;15(1):10393. doi: 10.1038/s41467-024-54741-w 39613754 PMC11607449

[13] GBD 2023 Demographics Collaborators. Global age-sex-specific all-cause mortality and life expectancy estimates for 204 countries and territories and 660 subnational locations, 1950-2023: a demographic analysis for the Global Burden of Disease Study 2023. Lancet. 2025;406:1731–810. doi: 10.1016/S0140-6736(25)01330-3 41092927 PMC12535839

[14] GBD 2023 Disease and Injury and Risk Factor Collaborators. Burden of 375 diseases and injuries, risk-attributable burden of 88 risk factors, and healthy life expectancy in 204 countries and territories, including 660 subnational locations, 1990-2023: a systematic analysis for the Global Burden of Disease Study 2023. Lancet. 2025;406:1873–922. doi: 10.1016/S0140-6736(25)01637-X 41092926 PMC12535840

[15] GBD 2021 Carbon Monoxide Poisoning Collaborators. Global, regional, and national mortality due to unintentional carbon monoxide poisoning, 2000-2021: results from the Global Burden of Disease Study 2021. Lancet Public Health. 2023;8(11):e839–49. doi: 10.1016/S2468-2667(23)00185-8 37813118 PMC10602911

[16] GBD 2023 Vaccine Coverage Collaborators. Global, regional, and national trends in routine childhood vaccination coverage from 1980 to 2023 with forecasts to 2030: a systematic analysis for the Global Burden of Disease Study 2023. Lancet. 2025;406:235–60. doi: 10.1016/S0140-6736(25)01037-2 40578370 PMC12338332

[17] Zhang N, Wu J, Wang Q, Liang Y, Li X, Chen G, et al. Global burden of hematologic malignancies and evolution patterns over the past 30 years. Blood Cancer J. 2023;13(1):82. doi: 10.1038/s41408-023-00853-3 37193689 PMC10188596

[18] Li X, Qu P, Yan P, Kou Y, Song J, Wu D, et al. Trends in burden and mortality of congenital birth defects in G20 countries (1990-2021) and predictions for 2022-2040. BMC Pregnancy Childbirth. 2025;25(1):494. doi: 10.1186/s12884-025-07617-w 40281466 PMC12023674

[19] Zhang N, Wang Q, Chen G, Liu L, Wang Z, Ma L, et al. Jab1 promotes immune evasion and progression in acute myeloid leukemia models under oxidative stress. J Clin Invest. 2025;135(20):e183761. doi: 10.1172/JCI183761 40763038 PMC12520683

[20] Wills NK, Singh N, Koegelenberg CFN, Allwood BW. Post-tuberculosis lung disease in people with HIV: a scoping and narrative review. Curr Opin Infect Dis. 2026;39(1):1–15. doi: 10.1097/QCO.0000000000001163 41452090

[21] Abbas U, Masood KI, Iqbal T, Jamil B, Qaiser S, Yameen M, et al. Individuals with latent tuberculosis in a high TB endemic country show mild COVID-19. PLoS One. 2025;20(12):e0339240. doi: 10.1371/journal.pone.0339240 41468475 PMC12753056

[22] LeBrun DG. CORR Insights: What Are the Mortality, Infection, and Nonunion Rates After Periprosthetic Femoral Fractures in the United States?. Clin Orthop Relat Res. 2024;482(3):484–6. doi: 10.1097/CORR.0000000000002893 37847412 PMC10871792

[23] Tu Y-R, Tsai T-Y, Lin M-S, Tu K-H, Lee C-C, Wu VC-C, et al. Association between initial dialytic modalities and the risks of mortality, infection death, and cardiovascular events: A nationwide population-based cohort study. Sci Rep. 2020;10(1):8066. doi: 10.1038/s41598-020-64986-2 32415125 PMC7229162

[24] Chutiyami M, Bello UM, Salihu D, Ndwiga D, Kolo MA, Maharaj R, et al. COVID-19 pandemic-related mortality, infection, symptoms, complications, comorbidities, and other aspects of physical health among healthcare workers globally: An umbrella review. Int J Nurs Stud. 2022;129:104211. doi: 10.1016/j.ijnurstu.2022.104211 35278750 PMC8855608

[25] Gao S, Huang X, Zhou X, Dai X, Han J, Chen Y, et al. A comprehensive evaluation of risk factors for mortality, infection and colonization associated with CRGNB in adult solid organ transplant recipients: a systematic review and meta-analysis. Ann Med. 2024;56(1):2314236. doi: 10.1080/07853890.2024.2314236 38442299 PMC10916923

[26] Tejamaya M, Putri AA, Widanarko B, Wirawan IMA, Kurniawan B, Thamrin Y. Dynamics of COVID-19 Risk Perception in Indonesia: A Consecutive Cross-sectional Study From 2020 to 2022. Saf Health Work. 2025;16(4):421–30. doi: 10.1016/j.shaw.2025.07.005 41477049 PMC12750300

[27] Arning N, Fryer HR, Wilson DJ. Identifying direct risk factors in UK Biobank via simultaneous Bayesian-frequentist model-averaged hypothesis testing using Doublethink. Proc Natl Acad Sci U S A. 2026;123(1):e2514138122. doi: 10.1073/pnas.2514138122 41481468 PMC12773712

[28] Ballif M, Banholzer N, Perrig L, Avihingsanon A, Nsonde DM, Obatsa S, et al. The long-term impact of the COVID-19 pandemic on tuberculosis care and infection control measures in anti-retroviral therapy (ART) clinics in low- and middle-income countries: a multiregional site survey in Asia and Africa. BMJ Glob Health. 2025;10(3):e017828. doi: 10.1136/bmjgh-2024-017828 40127942 PMC11934364

[29] Jin J, Xu Q, Zhang X, Zhu A, Xia W, Moog C, et al. HIV infection and immunosenescence: challenges and intervention strategies. BMC Med. 2025;24(1):8. doi: 10.1186/s12916-025-04545-6 41331594 PMC12777344

[30] Takeda K, Takazono T, Ide S, Yoshida M, Iwanaga N, Hosogaya N, et al. Clinical features of Mycobacterium abscessus complex and Mycobacterium kansasii pulmonary disease in Kyushu, Japan. Respir Investig. 2026;64(1):101358. doi: 10.1016/j.resinv.2025.101358 41455168

[31] Blakeslee-Carter J, Robinson S, Scali ST, Novak Z, Shahid Z, Huber TS, et al. Cause-specific long-term mortality after physician-modified branched/fenestrated endovascular aortic repair. J Vasc Surg. 2026;83(4):1048–55. doi: 10.1016/j.jvs.2025.10.100 41360189

[32] Jian B, Shen G, Liu J, Li W, Li Z, Tan K, et al. Clinical features, epidemiology, treatment, and prognosis of multicentre pediatric pneumococcal meningitis in China from 2019 to 2021. BMC Infect Dis. 2025;25(1):1739. doi: 10.1186/s12879-025-12049-0 41444504 PMC12729672

[33] Dawson WD, Stodola A, Carder P, Cellarius K, Smith L, Brandis L, et al. Supporting older-adult behavioral health: building the first state Center of Excellence for Behavioral Health and Aging. Health Aff Sch. 2025;3(9):qxaf175. doi: 10.1093/haschl/qxaf175 40979230 PMC12449125

[34] Yang X, Wang H, Chen S, He Y, Chen Y. Exploring the caregiving perceptions and needs of family caregivers of older stroke patients: a descriptive qualitative study. Int J Qual Stud Health Well-being. 2025;20(1):2588908. doi: 10.1080/17482631.2025.2588908 41292099 PMC12677149

[35] Zhang N, Zhang P, Chen Y, Lou S, Zeng H, Deng J. Clusterization in acute myeloid leukemia based on prognostic alternative splicing signature to reveal the clinical characteristics in the bone marrow microenvironment. Cell Biosci. 2020;10:118. doi: 10.1186/s13578-020-00481-5 33062256 PMC7552347

[36] Luvira V, Ngamprasertchai T, Pitisuttithum P. Pneumococcal conjugate vaccines in older adults and immunocompromised individuals. Expert Rev Vaccines. 2026;25(1):1–10. doi: 10.1080/14760584.2025.2602525 41368837

[37] Kumar M, Sundarsingh V, Ariharan K, Prashanth YM. Skull base osteomyelitis following Klebsiella pneumoniae meningitis in an immunocompromised host. BMJ Case Rep. 2025;18(12):e267719. doi: 10.1136/bcr-2025-267719 41475873

[38] GBD 2023 Causes of Death Collaborators. Global burden of 292 causes of death in 204 countries and territories and 660 subnational locations, 1990-2023: a systematic analysis for the Global Burden of Disease Study 2023. Lancet. 2025;406:1811–72. doi: 10.1016/S0140-6736(25)01917-8 41092928 PMC12535838

