# Supplementary material for: Spatiotemporal patterns of prevalence and mortality from respiratory infections and tuberculosis across Japan and its prefectures
Source: PLoS One. 2026 Jun 17;21(6):e0351936. doi: 10.1371/journal.pone.0351936 (PMC13274887; doi:10.1371/journal.pone.0351936)
Supplement: S1 Fig — (A) Percentage change in prevalence cases from 2010 to 2023.(B) Percentage change in death cases from 2010 to 2023.Maps were generated by the authors in R using publicly available administrative boundary data for Japan, without the use of Google Maps, satellite imagery, or proprietary basemaps. (PDF) [file pone.0351936.s001.pdf]

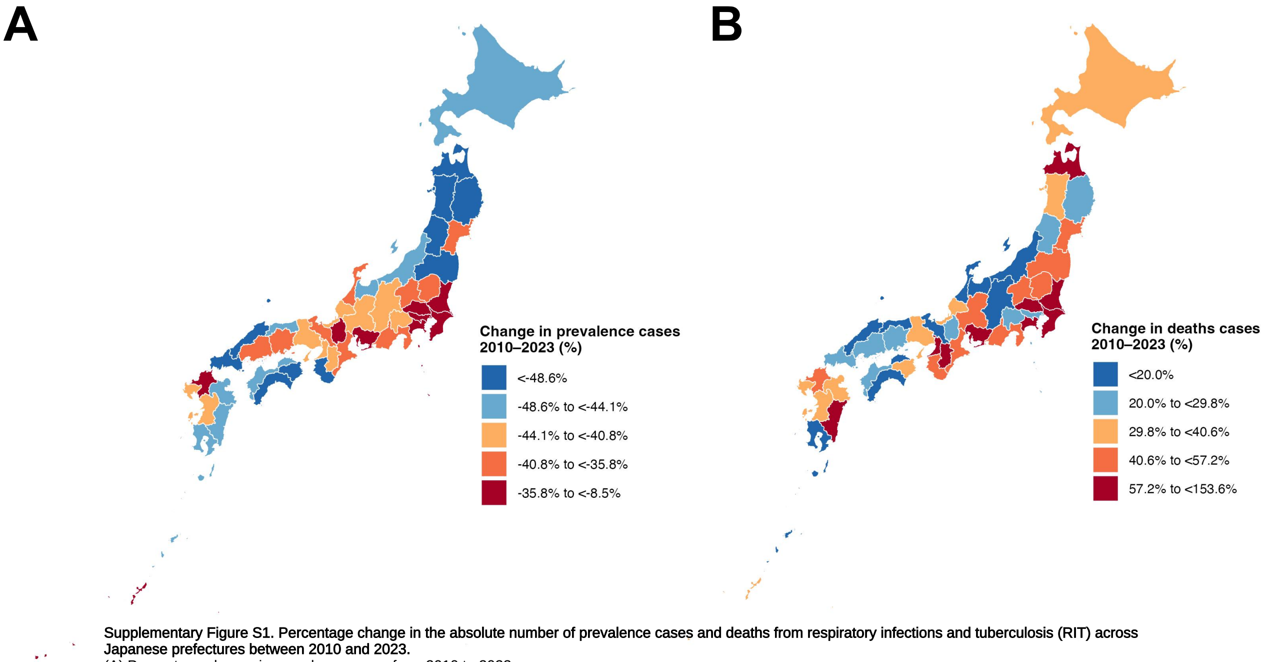

Supplementary Figure S1. Percentage change in the absolute number of prevalence cases and deaths from respiratory infections and tuberculosis (RIT) across Japanese prefectures between 2010 and 2023.
(A) Percentage change in prevalence cases from 2010 to 2023.
(B) Percentage change in death cases from 2010 to 2023.
