# Supplementary material for: Spatiotemporal patterns of prevalence and mortality from respiratory infections and tuberculosis across Japan and its prefectures
Source: PLoS One. 2026 Jun 17;21(6):e0351936. doi: 10.1371/journal.pone.0351936 (PMC13274887; doi:10.1371/journal.pone.0351936)
Supplement: Attachment — Submitted filename: Reviewer Report-plos one D-26-00936.pdf [file pone.0351936.s004.pdf]

Reviewer Report -PLOS ONE – D-26-00936

Manuscript Title

Spatiotemporal patterns of prevalence and mortality from respiratory infections and tuberculosis across Japan and its prefectures

---

General Assessment

This manuscript presents a comprehensive spatiotemporal analysis of respiratory infections and tuberculosis (RIT) burden in Japan from 2010–2023 using Global Burden of Disease (GBD) estimates. The study integrates national and prefectural trends in prevalence, mortality, demographic stratification, and etiology-specific mortality patterns.

Overall, the study is **methodologically sound**, clearly written, and aligned with *PLOS ONE*'s scope of publishing **robust, well-executed population health research**. The analysis addresses an important public health question in the context of population ageing and provides policy-relevant insights. However, **several issues need to be addressed before publication**, primarily concerning clarity of definitions, consistency of reporting, interpretation of GBD uncertainty, and presentation quality.

Major Comments

1. Clarity and Conceptual Framing of RIT as a Composite Outcome

Issue:
The manuscript defines RIT as a broad composite category including tuberculosis, upper and lower respiratory infections, otitis media, and COVID-19. While this follows GBD definitions, the **biological and epidemiological heterogeneity** of these conditions is substantial.

Concerns:

- Combining acute viral infections, bacterial pneumonias, tuberculosis, otitis media, and COVID-19 may obscure pathogen-specific trends and drivers.
- Interpretation of prevalence–mortality "decoupling" may differ substantially across components.

Recommendations:

- Explicitly justify the **use of a composite RIT construct** in the Introduction and Methods (beyond citing GBD conventions).
- In the Discussion, add a paragraph acknowledging how aggregation may mask divergent trends (e.g., TB vs. COVID-19 vs. pneumococcal disease).
- Consider adding a **supplementary sensitivity analysis** excluding COVID-19 deaths, given their strong influence post-2020.

---

2. Interpretation of Estimated Annual Percentage Change (EAPC)

Issue:
EAPC results are central to the manuscript, yet interpretation is occasionally inconsistent with confidence intervals.

Examples:

- Several EAPCs are reported as positive or negative despite **95% CIs crossing zero**, implying no statistically supported monotonic trend.
- Language such as "increased," "declined," or "upward trend" is sometimes used without caution.

Recommendations:

- Revise wording to distinguish **point estimates** from **statistically supported trends**.
- For EAPCs with CIs including zero, use neutral phrasing such as:

  "No clear long-term monotonic trend was observed."

- Consider adding a short explanatory sentence in the Methods clarifying how EAPC uncertainty should be interpreted.

---

3. Use and Communication of GBD Uncertainty

Issue:
While uncertainty intervals (UIs) are reported, their implications are not fully integrated into interpretation.

Concerns:

- Prefectural estimates, especially etiology-specific mortality, may have substantial uncertainty due to modeling.
- Readers may overinterpret small differences between regions or years.

Recommendations:

- Expand the **Limitations** section to more explicitly address:
  - Uncertainty propagation in subnational GBD estimates
  - Potential instability of prefectural-level etiology attribution
- Add a brief statement cautioning against overinterpretation of small regional differences.

---

4. Etiology-Specific Analysis: Strength but Needs Clearer Methods

Issue:
The etiology analysis is a major strength, but the **methodological description is insufficiently detailed**.

Concerns:

- It is unclear how GBD assigns cause-specific mortality within the RIT category.
- The distinction between "etiology of death" and "etiology of infection" is not always clear.

Recommendations:

- Expand the Etiology-specific analysis subsection to clarify:
  - Whether causes are mutually exclusive
  - How deaths were attributed when multiple infections were present
- Clarify that these are **modeled etiological attributions**, not laboratory-confirmed causes.

---

5. Discussion of COVID-19 Effects

Issue:
COVID-19 is included in RIT, but its impact is not sufficiently disentangled.

Concerns:

- The mortality rebound in 2021–2022 is plausibly driven by COVID-19.
- Readers may attribute changes to broader RIT dynamics rather than pandemic effects.

Recommendations:

- Add a **dedicated discussion paragraph** explicitly addressing:
  - COVID-19's contribution to mortality trends
  - How inclusion of COVID-19 influences long-term interpretation

- If possible, include a supplementary figure showing COVID-19's proportional contribution over time.

Minor Comments

1. Editorial and Formatting Issues

- Several typographical issues appear in tables (line breaks, truncated text, spacing errors).
- Age group descriptions are occasionally inconsistent (e.g., "75 years" vs "75+ years").

Recommendation:
Conduct a full editorial pass, particularly on **Tables 1 and 2**, to improve readability.

---

2. Abstract Clarity

- The abstract is dense and includes many numerical values, which may overwhelm readers.

Recommendation:
Slightly simplify numerical reporting and emphasize **directional trends and conclusions**.

---

3. Figure Legends

- Figure legends are informative but could better emphasize **key takeaways**.

Recommendation:
Add one short interpretative sentence per figure summarizing the main pattern.

---

4. Ethics Statement

- The ethics approval is clearly stated; however, use of publicly available GBD data typically does not require institutional review.

Recommendation:
Clarify whether ethics approval was required or simply obtained as institutional policy.

Strengths of the Study

- **Comprehensive temporal and spatial coverage** (2010–2023, all 47 prefectures)
- Rigorous use of standardized GBD methodology
- Clear stratification by age and sex
- Valuable examination of **etiological transitions**
- Strong relevance to ageing-society public health planning
- Aligns well with *PLOS ONE*'s emphasis on methodological rigor over novelty

---

Overall Recommendation

The manuscript is scientifically solid and well within the scope of *PLOS ONE*, but it requires clarification and refinement in interpretation, transparency regarding GBD uncertainty, and improved presentation. Addressing the comments above will substantially strengthen the manuscript's clarity, credibility, and policy relevance.
