# Supplementary material for: Spatiotemporal patterns of prevalence and mortality from respiratory infections and tuberculosis across Japan and its prefectures
Source: PLoS One. 2026 Jun 17;21(6):e0351936. doi: 10.1371/journal.pone.0351936 (PMC13274887; doi:10.1371/journal.pone.0351936)
Supplement: Attachment — Submitted filename: Response to Reviewers.docx [file pone.0351936.s006.docx]

**Author_Response_Letter (Manuscript ID: PONE-D-26-00936)**

Dear Editors and Reviewers:

We would like to thank the editors and reviewers’ work devoted to our manuscript entitled "Spatiotemporal patterns of prevalence and mortality from respiratory infections and tuberculosis across Japan and its prefectures" (Manuscript ID: PONE-D-26-00936). Those comments are all valuable and very helpful for revising and improving our paper. We have studied the comments carefully and made corrections that we hope to meet with approval.

Each concern is discussed in detail below. These revisions were performed to address the reviewers’ concerns. Thank you again for allowing us to resubmit our manuscript for your consideration.

**Responses to Editorial Requirements**

Journal Requirements:

When submitting your revision, we need you to address these additional requirements.

1. Please ensure that your manuscript meets PLOS ONE's style requirements, including those for file naming. The PLOS ONE style templates can be found at

https://journals.plos.org/plosone/s/file?id=wjVg/PLOSOne_formatting_sample_main_body.pdf and https://journals.plos.org/plosone/s/file?id=ba62/PLOSOne_formatting_sample_title_authors_affiliations.pdf

**Response:** Thank you for this comment. We have carefully revised the manuscript according to the PLOS ONE formatting and file-naming requirements, including the title page, manuscript structure, tables, and figure formatting.

2. Please provide a complete Data Availability Statement in the submission form, ensuring you include all necessary access information or a reason for why you are unable to make your data freely accessible. If your research concerns only data provided within your submission, please write "All data are in the manuscript and/or supporting information files" as your Data Availability Statement.

**Response:** Thank you for this comment. We have added the required Data Availability Statement in the revised manuscript and submission form: “All data are in the manuscript and/or supporting information files.”

3. We note that Figure 3 and Figure S1 in your submission contain [map/satellite] images which may be copyrighted. All PLOS content is published under the Creative Commons Attribution License (CC BY 4.0), which means that the manuscript, images, and Supporting Information files will be freely available online, and any third party is permitted to access, download, copy, distribute, and use these materials in any way, even commercially, with proper attribution. For these reasons, we cannot publish previously copyrighted maps or satellite images created using proprietary data, such as Google software (Google Maps, Street View, and Earth). For more information, see our copyright guidelines: http://journals.plos.org/plosone/s/licenses-and-copyright.

We require you to either (1) present written permission from the copyright holder to publish these figures specifically under the CC BY 4.0 license, or (2) remove the figures from your submission:

1. You may seek permission from the original copyright holder of Figure 3 and Figure S1 to publish the content specifically under the CC BY 4.0 license.

We recommend that you contact the original copyright holder with the Content Permission Form (http://journals.plos.org/plosone/s/file?id=7c09/content-permission-form.pdf) and the following text:

“I request permission for the open-access journal PLOS ONE to publish XXX under the Creative Commons Attribution License (CCAL) CC BY 4.0 (http://creativecommons.org/licenses/by/4.0/). Please be aware that this license allows unrestricted use and distribution, even commercially, by third parties. Please reply and provide explicit written permission to publish XXX under a CC BY license and complete the attached form.”

Please upload the completed Content Permission Form or other proof of granted permissions as an ""Other"" file with your submission.

In the figure caption of the copyrighted figure, please include the following text: “Reprinted from [ref] under a CC BY license, with permission from [name of publisher], original copyright [original copyright year].”

2. If you are unable to obtain permission from the original copyright holder to publish these figures under the CC BY 4.0 license or if the copyright holder’s requirements are incompatible with the CC BY 4.0 license, please either i) remove the figure or ii) supply a replacement figure that complies with the CC BY 4.0 license. Please check copyright information on all replacement figures and update the figure caption with source information. If applicable, please specify in the figure caption text when a figure is similar but not identical to the original image and is therefore for illustrative purposes only.

The following resources for replacing copyrighted map figures may be helpful:

USGS National Map Viewer (public domain): http://viewer.nationalmap.gov/viewer/

The Gateway to Astronaut Photography of Earth (public domain): http://eol.jsc.nasa.gov/sseop/clickmap/Maps at the CIA (public domain): https://www.cia.gov/library/publications/the-world-factbook/index.html and https://www.cia.gov/library/publications/cia-maps-publications/index.html

NASA Earth Observatory (public domain): http://earthobservatory.nasa.gov/

Landsat: http://landsat.visibleearth.nasa.gov/

USGS EROS (Earth Resources Observatory and Science (EROS) Center) (public domain): http://eros.usgs.gov/#

Natural Earth (public domain): http://www.naturalearthdata.com/

**Response:** Thank you for this comment. We have regenerated Figure 3 and Figure S1 using an R-based workflow with publicly available administrative boundary data for Japan obtained through the geodata package (GADM level-1 boundaries). The maps were generated using R packages including sf, ggplot2, and geodata. No Google Maps, Google Earth, satellite imagery, or proprietary basemap was used; therefore, the revised figures do not involve copyrighted map or satellite image content.

The following text has been added to the Methods section (Statistical analysis subsection):

*“All data management, analyses, visualizations, and map generation were performed in R (version 4.2.2; R Foundation for Statistical Computing, Vienna, Austria). Prefecture-level maps were generated using publicly available administrative boundary data for Japan obtained through the geodata package (GADM level-1 boundaries), without the use of proprietary basemaps or satellite imagery.”*

The following statement has also been added to the captions of Figure 3:

*“Maps were generated by the authors in R using publicly available administrative boundary data for Japan.”*

4. Please include captions for your Supporting Information files at the end of your manuscript, and update any in-text citations to match accordingly. Please see our Supporting Information guidelines for more information: http://journals.plos.org/plosone/s/supporting-information.

**Response:** We have added captions for all Supporting Information files at the end of the revised manuscript.

5. If the reviewer comments include a recommendation to cite specific previously published works, please review and evaluate these publications to determine whether they are relevant and should be cited. There is no requirement to cite these works unless the editor has indicated otherwise.

**Response:** Thank you. No reviewers requested citation of additional specific references.

**Responses to Reviewers**

1. Is the manuscript technically sound, and do the data support the conclusions?

The manuscript must describe a technically sound piece of scientific research with data that supports the conclusions. Experiments must have been conducted rigorously, with appropriate controls, replication, and sample sizes. The conclusions must be drawn appropriately based on the data presented.

Reviewer #1: Yes

Reviewer #2: Partly

2. Has the statistical analysis been performed appropriately and rigorously?

Reviewer #1: Yes

Reviewer #2: No

3. Have the authors made all data underlying the findings in their manuscript fully available?

The PLOS Data policy requires authors to make all data underlying the findings described in their manuscript fully available without restriction, with rare exception (please refer to the Data Availability Statement in the manuscript PDF file). The data should be provided as part of the manuscript or its supporting information, or deposited to a public repository. For example, in addition to summary statistics, the data points behind means, medians and variance measures should be available. If there are restrictions on publicly sharing data—e.g. participant privacy or use of data from a third party—those must be specified.

Reviewer #1: Yes

Reviewer #2: Yes

4. Is the manuscript presented in an intelligible fashion and written in standard English?

PLOS ONE does not copyedit accepted manuscripts, so the language in submitted articles must be clear, correct, and unambiguous. Any typographical or grammatical errors should be corrected at revision, so please note any specific errors here.

Reviewer #1: Yes

Reviewer #2: Yes

5. Review Comments to the Author

Please use the space provided to explain your answers to the questions above. You may also include additional comments for the author, including concerns about dual publication, research ethics, or publication ethics. (Please upload your review as an attachment if it exceeds 20,000 characters)

**Response:** We sincerely thank the reviewers for their valuable comments and suggestions. The manuscript has been carefully revised accordingly, and all revisions are detailed below.

**Responses to Reviewer #1**

Reviewer #1: The research article meets the criteria.

1. The source of the data used has been clearly explained

2. Legible and standard English has been used

3. Ethical considerations and protocol were followed

4. The study provides new insights for the scientific community

All in all, I believe that the article is fit for publication.

**Response:** We sincerely thank the reviewer for the positive evaluation and strong support of our work. We greatly appreciate the reviewer’s recognition of the methodological rigor, ethical compliance, and public health relevance of our study.

**Responses to Reviewer #1**

Reviewer #2: General Assessment

This manuscript presents a comprehensive spatiotemporal analysis of respiratory infections and tuberculosis (RIT) burden in Japan from 2010–2023 using Global Burden of Disease (GBD) estimates. The study integrates national and prefectural trends in prevalence, mortality, demographic stratification, and etiology-specific mortality patterns.

Overall, the study is methodologically sound, clearly written, and aligned with PLOS ONE’s scope of publishing robust, well-executed population health research. The analysis addresses an important public health question in the context of population ageing and provides policy-relevant insights. However, several issues need to be addressed before publication, primarily concerning clarity of definitions, consistency of reporting, interpretation of GBD uncertainty, and presentation quality.

see the attached filed for detailed report

**Response:** We sincerely thank the reviewer for the thoughtful and constructive comments. We have carefully revised the manuscript according to the comments in the attached detailed report. Detailed point-by-point responses are provided below.

Major Comments

1. Clarity and Conceptual Framing of RIT as a Composite Outcome

Issue:The manuscript defines RIT as a broad composite category including tuberculosis, upper and lower respiratory infections, otitis media, and COVID-19. While this follows GBD definitions, the biological and epidemiological heterogeneity of these conditions is substantial.

Concerns:

• Combining acute viral infections, bacterial pneumonias, tuberculosis, otitis media, and COVID-19 may obscure pathogen-specific trends and drivers.

• Interpretation of prevalence-mortality "decoupling" may differ substantially across components.

Recommendations:

• Explicitly justify the use of a composite RIT construct in the Introduction and Methods (beyond citing GBD conventions).

• In the Discussion, add a paragraph acknowledging how aggregation may mask divergent trends (e.g., TB vs. COVID-19 vs. pneumococcal disease).

• Consider adding a supplementary sensitivity analysis excluding COVID-19 deaths, given their strong influence post-2020.

**Response:** Thank you for this important comment. In response, we further clarified the rationale for using the composite RIT construct in the **Introduction** section. The following text was added:

*“Within the GBD framework, RIT represents a standardized composite category integrating multiple major respiratory infectious conditions across populations and demographic groups. Although these conditions differ biologically and epidemiologically, evaluating them collectively may better reflect the overall healthcare and mortality burden imposed by respiratory infectious diseases in rapidly ageing societies such as Japan.”*

In addition, we expanded the **Discussion** section to acknowledge the limitations of aggregating heterogeneous respiratory infectious diseases and the potential impact of COVID-19 on temporal trends. The following text was added:

*“In addition, because RIT was analyzed as a composite category, aggregation of biologically heterogeneous conditions may mask pathogen-specific temporal patterns and epidemiological drivers. Furthermore, inclusion of COVID-19 within the standardized GBD framework likely contributed substantially to the mortality rebound observed after 2020 and may have influenced the observed prevalence–mortality divergence.”*

2. Interpretation of Estimated Annual Percentage Change (EAPC)

Issue:EAPC results are central to the manuscript, yet interpretation is occasionally inconsistent with confidence intervals.

Examples:

• Several EAPCs are reported as positive or negative despite 95% Cls crossing zero, implying

no statistically supported monotonic trend

• Language such as "increased," "declined," or "upward trend" is sometimes used without caution.

Recommendations:

• Revise wording to distinguish point estimates from statistically supported trends.

• For EAPCs with Cls including zero, use neutral phrasing such as:

"No clear long-term monotonic trend was observed."

• Consider adding a short explanatory sentence in the Methods clarifying how EAPC uncertainty should be interpreted.

**Response:** Thank you for this important comment. In response, we added an explanatory statement in the “Temporal trend analysis” subsection of the **Methods** section to clarify interpretation of EAPC uncertainty. The following text was added:

*“EAPC estimates with 95% confidence intervals crossing zero were interpreted as indicating no clear long-term monotonic trend.”*

In addition, we revised several statements in the Results section to distinguish point estimates from statistically supported temporal trends. For EAPCs with confidence intervals including zero, more neutral wording such as *“no clear long-term monotonic trend was observed”* was adopted accordingly.

3. Use and Communication of GBD Uncertainty

Issue:While uncertainty intervals (Uls) are reported, their implications are not fully integrated into interpretation.

Concerns:

• Prefectural estimates, especially etiology-specific mortality, may have substantial uncertainty due to modeling.

• Readers may overinterpret small differences between regions or years.

Recommendations:

• Expand the Limitations section to more explicitly address:

• Uncertainty propagation in subnational GBD estimates

• Potential instability of prefectural-level etiology attribution

• Add a brief statement cautioning against overinterpretation of small regional differences.

**Response:** Thank you for this important comment. In response, we expanded the Limitations section in the Discussion to further address uncertainty in GBD-derived estimates, particularly at the prefectural and etiology-specific levels. The following text was added:

*“Etiology-specific patterns depend on cause-of-death attribution, diagnostic practices, and GBD modeling assumptions, which may vary across regions and over time and may introduce additional uncertainty in prefectural-level etiological estimates.”*

In addition, we further clarified that etiology-specific patterns may be influenced by variability in cause-of-death attribution and diagnostic practices across regions and over time.

4. Etiology-Specific Analysis: Strength but Needs Clearer Methods

Issue:The etiology analysis is a major strength, but the methodological description is insufficiently detailed.

Concerns:

• It is unclear how GBD assigns cause-specific mortality within the RIT category.

• The distinction between "etiology of death" and "etiology of infection" is not always clear.

Recommendations:

• Expand the Etiology-specific analysis subsection to clarify:

• Whether causes are mutually exclusive

• How deaths were attributed when multiple infections were present

• Clarify that these are modeled etiological attributions, not laboratory-confirmed causes.

**Response:** Thank you for this important comment. In response, we expanded the “Etiology-specific analysis” subsection in the Methods section to further clarify the interpretation of etiology-specific estimates within the GBD framework. The following text was added:

*“Etiological categories in the GBD framework represent modeled cause-specific attributions rather than laboratory-confirmed infections. Causes were treated as mutually exclusive within the GBD estimation framework, and deaths involving multiple potential infectious contributors were assigned according to probabilistic cause attribution models used by the GBD study.”*

5. Discussion of COVID-19 Effects

Issue:COVID-19 is included in RIT, but its impact is not sufficiently disentangled.

Concerns:

• The mortality rebound in 2021-2022 is plausibly driven by COVID-19.

• Readers may attribute changes to broader RIT dynamics rather than pandemic effects.

Recommendations:

• Add a dedicated discussion paragraph explicitly addressing:

• COVID-19's contribution to mortality trends

• How inclusion of COVID-19 influences long-term interpretation

• If possible, include a supplementary figure showing COVID-19's proportional contribution over time.

**Response:** Thank you for this important comment. In response, we expanded the **Discussion** section to further clarify the contribution of COVID-19 to the mortality rebound observed during 2021–2022 and its influence on the interpretation of long-term RIT trends. The following text was added:

*“Furthermore, inclusion of COVID-19 within the standardized GBD framework likely contributed substantially to the mortality rebound observed after 2020 and may have influenced the observed prevalence–mortality divergence.”*

Minor Comments

1. Editorial and Formatting Issues

• Several typographical issues appear in tables (line breaks, truncated text, spacing errors).

• Age group descriptions are occasionally inconsistent (e.g., "75 years" vs "75+ years").

Recommendation:

Conduct a full editorial pass, particularly on Tables 1 and 2, to improve readability.

**Response**: Thank you for this comment. We conducted a full editorial check of the manuscript and revised Tables 1 and 2 to correct typographical issues, line breaks, spacing errors, and inconsistent age-group descriptions. The age group “75+ years” has been standardized throughout the manuscript.

2. Abstract Clarity

• The abstract is dense and includes many numerical values, which may overwhelm readers.

Recommendation:

Slightly simplify numerical reporting and emphasize directional trends and conclusions.

**Response:** Thank you for this helpful suggestion. We revised the Abstract to simplify numerical reporting and improve readability by placing greater emphasis on the overall temporal trends, geographic disparities, and major conclusions of the study.

3. Figure Legends

• Figure legends are informative but could better emphasize key takeaways.

Recommendation:

Add one short interpretative sentence per figure summarizing the main pattern.

**Response:** We added brief interpretative summary statements to the figure legends to better highlight the main findings of each figure.

4. Ethics Statement

• The ethics approval is clearly stated; however, use of publicly available GBD data typically does not require institutional review.

Recommendation:

Clarify whether ethics approval was required or simply obtained as institutional policy.

Strengths of the Study

Comprehensive temporal and spatial coverage (2010-2023, all 47 prefectures)

• Rigorous use of standardized GBD methodology

• Clear stratification by age and sex

• Valuable examination of etiological transitions

Strong relevance to ageing-society public health planning

Aligns well with PLOS ONE's emphasis on methodological rigor over novelty

**Response:** Thank you for noting this important point. The manuscript already includes an Ethics statement clarifying that the study was based on publicly available and de-identified GBD data.

Overall Recommendation

The manuscript is scientifically solid and well within the scope of PLOS ONE, but it requires clarification and refinement in interpretation, transparency regarding GBD uncertainty, and improved presentation. Addressing the comments above will substantially strengthen the manuscript's clarity, credibility, and policy relevance.

**Response:** We sincerely thank the reviewer for the constructive comments and positive overall evaluation of our study.

6. PLOS authors have the option to publish the peer review history of their article (what does this mean?). If published, this will include your full peer review and any attached files.

If you choose “no”, your identity will remain anonymous but your review may still be made public.

Do you want your identity to be public for this peer review? For information about this choice, including consent withdrawal, please see our Privacy Policy.

Reviewer #1: No

Reviewer #2: Yes: Christopher F Njeh, PHD, FAAPM

**Response:** Thank you for the valuable feedback.

In addition, to improve the overall coherence of the manuscript, we made several adjustments throughout the text. Although we did not use track changes, all modifications have been carefully incorporated into the revised version.

We appreciate for Editors/Reviewers’ warm work earnestly and hope that the correction will meet with approval.

Once again, thank you very much for your comments and suggestion.

With best regards,

Zhimin Ding, M.D., Ph.D.

Department of Infectious Diseases

Dazhou Central Hospital
